# Macrophage migration inhibitory factor regulates TLR4 expression and modulates TCR/CD3-mediated activation in CD4+ T lymphocytes

**DOI:** 10.1038/s41598-019-45260-6

**Published:** 2019-06-28

**Authors:** Mohamed Alibashe-Ahmed, Thierry Roger, Veronique Serre-Beinier, Ekaterine Berishvili, Walter Reith, Domenico Bosco, Thierry Berney

**Affiliations:** 1 Cell Isolation and Transplantation Center, Department of Surgery, Geneva University Hospitals and University of Geneva, Geneva, Switzerland; 2 Unit of Thoracic and Endocrine Surgery, Department of Surgery, Geneva University Hospitals and University of Geneva, Geneva, Switzerland; 3 Infectious Diseases Service, Department of Medicine, Lausanne University Hospital, Epalinges, Switzerland; 4 Department of Pathology and Immunology, University of Geneva, 1211, Geneva, 4 Switzerland

**Keywords:** Cell death and immune response, Immunology

## Abstract

Toll-like receptor 4 (TLR4) is involved in CD4+ T lymphocyte-mediated pathologies. Here, we demonstrate that CD4+ T lymphocytes express functional TLR4 that contributes to their activation, proliferation and cytokine secretion. In addition, we demonstrate that TLR4-induced responses are mediated by macrophage migration inhibitory factor (MIF), a pro-inflammatory cytokine. We also demonstrate that MIF regulates suboptimal TCR/CD3-mediated activation of T lymphocytes. On one hand, MIF prevents excessive TCR/CD3-mediated activation of CD4+ T lymphocytes under suboptimal stimulation conditions and, on the other hand, MIF enables activated CD4+ T lymphocytes to sense their microenvironment and adapt their effector response through TLR4. Therefore, MIF appears to be a major regulator of the activation of CD4+ T lymphocytes and the intensity of their effector response. TLR4-mediated activation is thus an important process for T cell-mediated immunity.

## Introduction

In the last ten years, research in the field of autoimmunity and inflammatory diseases has provided ample support for the involvement of Toll-like receptors (TLRs) in T lymphocyte-mediated autoimmune or inflammatory pathologies^1,2^. TLRs and their signal transducer MyD88 are expressed in murine T lymphocytes^3,4^. TLR ligands are involved in the activation of effector T lymphocytes, either directly by binding to T lymphocyte expressed TLRs or via an indirect mechanism through TLR-engagement on innate immune cells. TLR4 is one of the best characterized members of the TLR family. TLR4 has been shown to be expressed by mouse and human CD4+ T lymphocytes, suggesting an evolutionary conserved function^3,5^. Although TLR4 expression has been described in both naïve and activated CD4+ T lymphocytes, its role in effector CD4+ T lymphocytes remains largely unknown^1–3,5–8^. In naïve CD4+ T lymphocytes, TLR4 could induce a costimulatory signal important for their activation, as observed in TLR9-induced activation of naïve CD4+ T lymphocytes^9^. Caramalho et *al*. reported that the triggering of TLR4 by lipopolysaccharide (LPS) induces a stronger proliferation of regulatory T cells (Tregs) than stimulation by IL-2, and that the simultaneous exposure to IL-2 and LPS potentiates the proliferation and regulatory function of Tregs. Reynolds et *al*. studied the role of TLR4 expressed by CD4+ T lymphocytes in the pathogenesis of experimental autoimmune encephalomyelitis (EAE), using a model of T lymphocyte transfer^2^. Compared to recipients of WT CD4+ T lymphocytes, recipients of TLR4-deficient CD4+ T lymphocytes exhibited virtually no clinical signs of EAE and expressed significantly reduced production of pro-inflammatory cytokines such as IL-17 and IFNγ. TLR4 signalling in CD4+ T lymphocytes seems to have no effect on T-cell polarization towards a Th1 or Th17 phenotype, but rather plays an important role in CD4+ T lymphocyte survival. However, in a model of experimental colitis, Gonzalez-Navajas et *al*. demonstrated that TLR4-deficient mice have exacerbated colitis^1^. In this model, TLR4 signalling in CD4+ T lymphocytes decreases the secretion of IFNγ through inhibition of the ERK1/2 MAPK signalling pathway and regulates the activation of TCR signaling by inducing MAPK phosphatases. The absence of a consensus on the role of TLR4 in T lymphocytes raises the question of why an evolutionary conserved receptor is expressed on CD4+ T lymphocytes.

Macrophage migration inhibitory factor (MIF) was characterized more than 50 years ago as a factor that is abundantly released by T cells and inhibited the random migration of peritoneal exudate cells^10^. Numerous cell types including immune, epithelial and endocrine cells constitutively express MIF^11^. Extracellular MIF acts through a receptor complex made of CD74 with or without CD44, CXCR2 and CXCR4 to initiate intracellular signalling^12–14^. Alternatively, MIF can directly interact with p53, JAB-1 or ribosomal protein S19 to modulate intracellular signalling^15–17^. Because of these interactions, MIF stimulates MAPK and PI3K/Akt signalling pathways, inhibits MAPK phosphatase-1 and modulates AP-1 activity to promote cell survival, cell proliferation and inflammatory processes^15–18^. Interestingly, MIF positively regulates the expression of TLR4 in macrophages^19^. Owing to its functions, MIF is an important regulator of innate immune responses and essential for fighting bacterial, viral and parasitic infections^20^. MIF has been implicated in the pathogenesis of multiple inflammatory and autoimmune diseases^11,21^. Curiously however, little is known about the role of MIF in modulating T cell functions. Here, we investigated whether TLR4 activation impacts on the effector functions of CD4+ T lymphocytes, and whether MIF could be involved in TLR4-mediated activation of CD4+ T lymphocytes.

We demonstrate that CD4+ T lymphocytes respond directly to LPS by increasing their effector functions under suboptimal TCR/CD3-mediated activation conditions, and that MIF is indispensable for LPS-induced activation of CD4+ T lymphocytes. Moreover, we demonstrate that MIF regulates TCR/CD3-mediated activation and prevents excessive lymphocyte activation under suboptimal TCR/CD3-mediated activation conditions. Therefore, MIF can be considered as a major regulator and rheostat of TCR/CD3− and TLR4− mediated activation of CD4+ T lymphocytes.

## Results

### LPS increases the effector functions of T lymphocytes in the absence of antigen-presenting cells

**Figure 1:**
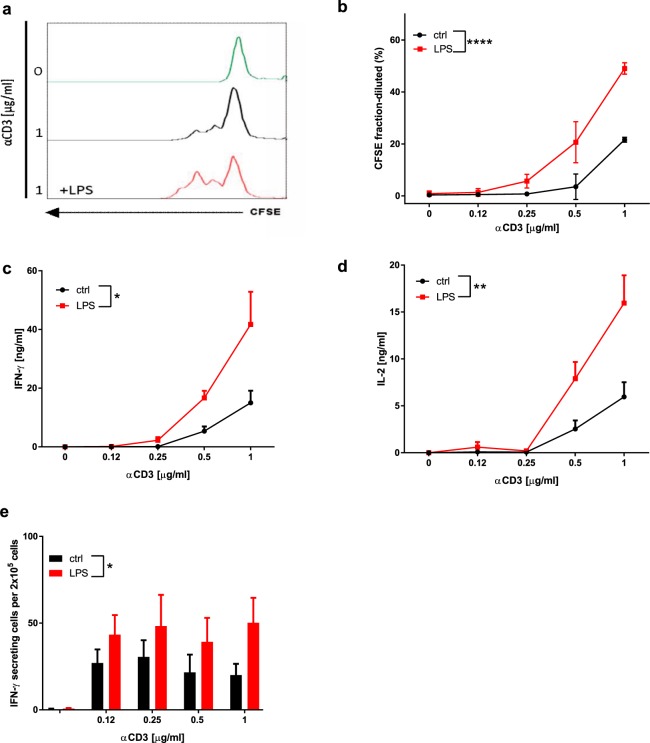
LPS increases the activation of BDC2.5 T lymphocytes in the absence of APC: (**a**) Representative images of CFSE fluorescence of unstimulated T lymphocytes (green) and T lymphocytes exposed to 1 μg/ml of anti-CD3 antibody in the presence (red) or absence (black) of LPS. (**b**) Proliferation was assessed by examining CFSE dilution of T lymphocytes exposed to increasing concentrations of anti-CD3 antibodies and 1 μg/ml anti-CD28 in the presence (red) or the absence (black) of LPS. (**c**) IFNγ concentrations in supernatants were determined after LPS stimulation (red). (**d**) IL-2 secretion was determined in the presence (red) or absence (black) of LPS. (**e**) Quantification by ELISPOT of IFNγ-producing cells among spleen lymphocytes exposed to increasing concentrations of anti-CD3 antibodies in the presence (red) or the absence (black) of LPS. Two-way ANOVA with Sidak post-test was used (**b**–**e**). Data are means ± SEM from three (**b**), four (**d**) and five experiments (**c**,**e**) *p ≤ 0.05, **p ≤ 0.01, ****p < 0.0001.

We assessed the impact of TLR4 activation on purified CD3+ T lymphocytes, in the absence of antigen-presenting cells (APCs). Isolated T lymphocytes were cultured for 48 hours with or without 1 μg/ml LPS in the presence or absence of increasing concentrations of anti-CD3 antibody and 1 μg/ml anti-CD28. Cell proliferation was assessed by means of CFSE dilution (Fig. 1a,b), while IL-2 and IFNγ production was measured by ELISA (Fig. 1c,d). In the absence of anti-CD3/CD28 stimulation, proliferation and cytokine production were undetectable, independently of the presence or absence of LPS (Fig. 1a–d). In contrast, LPS significantly enhanced anti-CD3/CD28 antibody-induced T lymphocyte proliferation (Fig. 1a,b), IL-2 and IFNγ secretion (Fig. 1c,d), and the number of IFNγ-producing T lymphocytes (Figs 1e and S1).

### LPS increases the activation of CD4+ T lymphocytes

**Figure 2:**
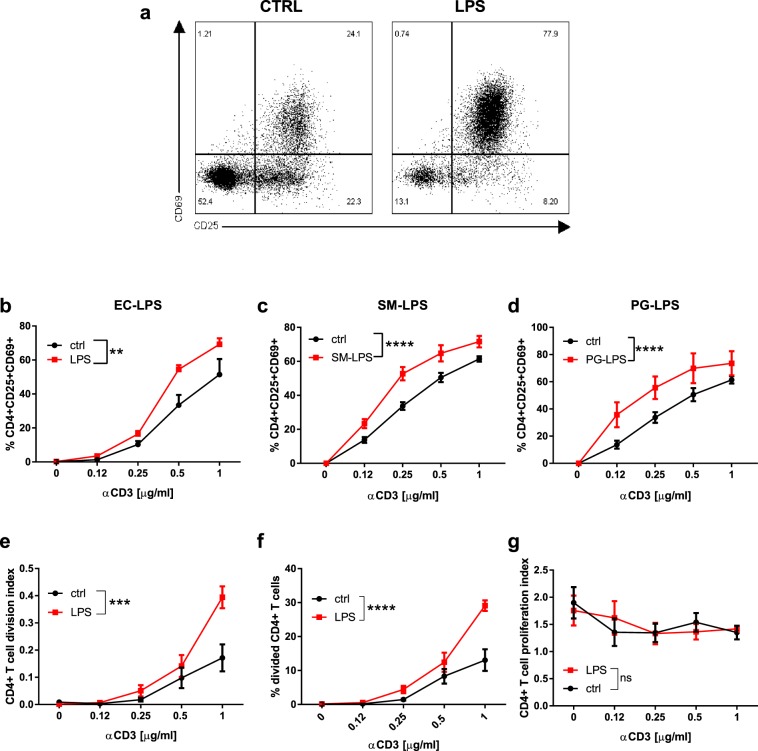
LPS directly increases the activation of BDC2.5 CD4+ T lymphocytes: (**a**) Representative image of anti-CD3/CD28 activated CD4+ T lymphocytes after stimulation with or without LPS. (**b**–**d**) quantification of CD69+CD25+CD4 + lymphocytes after stimulation with LPS from *E.coli* O111:B4 (EC-LPS), Salmonella Minnesota R595 (SM-LPS) or Porphyris Gingivalis (PG-LPS). (**e**–**g**) CD4+ T lymphocyte division and proliferation indexes, and percentages of divided CD4+ T lymphocytes, in presence (red) or absence (black) of LPS were measured by CFSE-dilution. Two-way ANOVA with Sidak post-test was used (**b**–**g**). Data are means ± SEM from four (c and d) and five experiments (**b**,**e**–**g**). ns = not significant **p ≤ 0.01, ***p ≤ 0.001, ****p < 0.0001.

**Figure 3:**
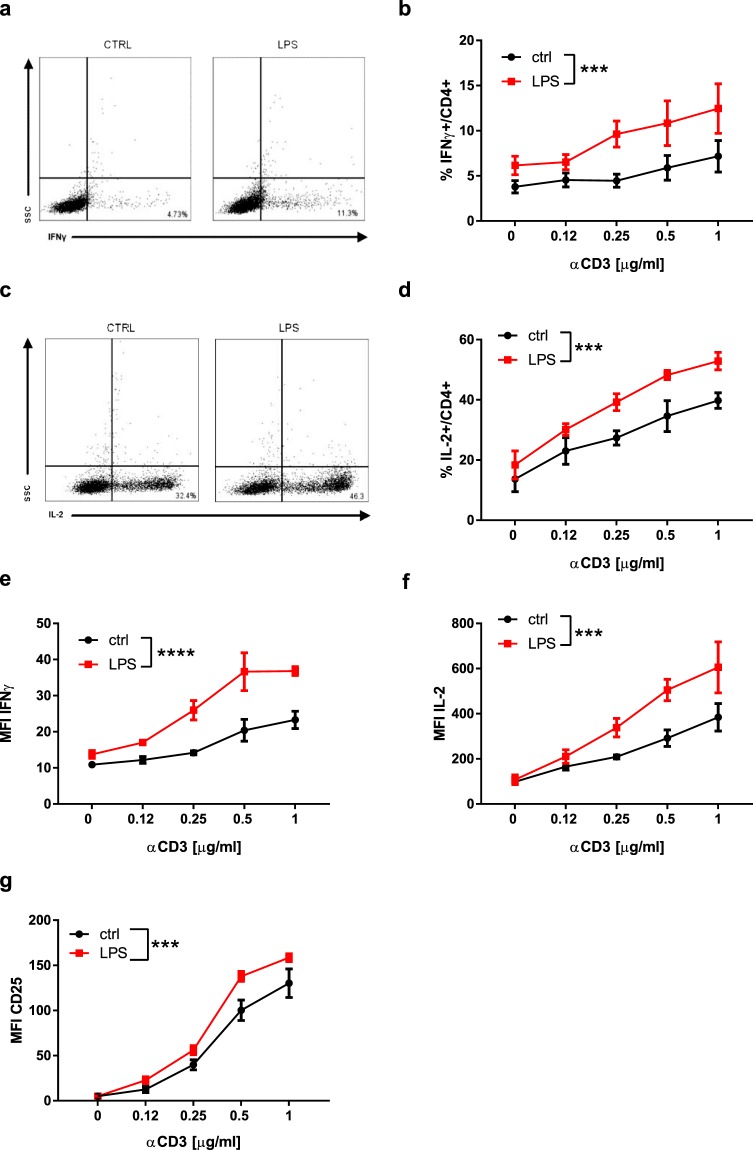
LPS increases the effector function of BDC2.5 CD4+ T lymphocytes: (**a**,**b**) Representative image and percentages of IFNγ-producing CD4+ T cells. (**c**,**d**) Representative image and percentages of IL-2-producing CD4+ T cells. (**e**,**f**) Quantification of IFNγ and IL-2 production by CD4+ T cells. (**g**) Quantification of CD25 expression in the presence or absence of LPS. Two-way ANOVA with Sidak post-test was used (**b**,**d,e–g**). Data are means ± SEM from three experiments. ***p ≤ 0.001, ****p < 0.0001.

We explored the effects of LPS on the activation of purified CD4+ T lymphocytes (Fig. 2). FITC-coupled LPS stained CD4+ T lymphocytes even though the shift of fluorescence measured by flow cytometry was small, suggesting that LPS interacted directly with CD4+ T lymphocytes (Fig. S2), and elicited a dose-dependent activation of CD4+ T lymphocytes, with a maximum effect at 1 μg/ml (not shown). We next assessed the effect of 1 μg/ml LPS on anti-CD3/CD28-induced expression of CD25, the high-affinity receptor for IL-2, and CD69 on CD4+ T lymphocytes (Fig. 2a,b). LPS-enhanced activation of CD4+ T lymphocytes was dependent on TCR/CD3 activation and maximal under suboptimal stimulation with 0.25–1 µg/ml of anti-CD3 antibody (Fig. 2b). To determine whether the LPS-induced effect was restricted to LPS from *Escherichia coli* we tested the effect of other ultrapure LPS preprations. LPS from *Porphyromonas gingivalis* and *Salmonella minnesota* R595 increased the activation of CD4+ T lymphocytes to a similar extent as LPS from *E.coli* O111:B4 (Fig. 2c,d). In addition, LPS-enhanced CD4+ T lymphocyte activation was not dependent on the genetic background, as similar effects of LPS were observed using lymphocytes isolated from C57BL/6, BALB/c and NOD mice (not shown). CFSE dilution was used to determine the effect of LPS on the proliferation of CD4+ T lymphocytes (Fig. 2e–g). Cell division and proliferation indexes, and percentages of divided cells in anti-CD3/CD28 activated CD4+ T lymphocytes were measured in the absence (control) or presence of LPS. Compared to control cells, LPS increased the division index of CD4+ T lymphocytes (Fig. 2e) and the percentage of CD4+ T lymphocytes that entered cell division (Fig. 2f). The proliferation index was not affected by LPS (Fig. 2g). LPS-induced CD4+ T cell proliferation was maximal upon stimulation with 1 μg/ml anti-CD3 antibody. In addition, LPS increased the number of IFNγ- and IL-2-expressing CD4+ T lymphocytes (Fig. 3a–d) and the production of IFNγ and IL-2 (Fig. 3e,f). It also increased the expression of CD25 (Fig. 3g). Taken together, these data confirm that LPS increases the activation and effector functions of CD4+ T lymphocytes.

### TLR4 mediates LPS-induced activation of CD4+ T cells

**Figure 4:**
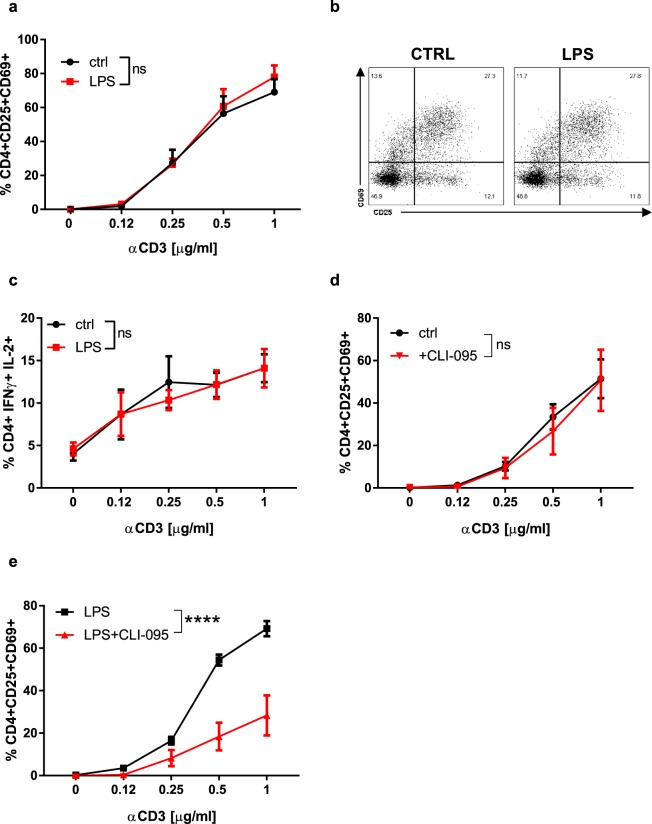
TLR4 mediates LPS-induced activation of CD4+ T lymphocytes: (**a**) Quantification of the number of activated TLR4^−/−^ CD4+ T lymphocytes in the presence of LPS. (**b**) Representative image of the effect of LPS on the activation of TLR4^−/−^ CD4+ T cells. (**c**) Quantification of the numbers of IFNγ- and IL-2- producing TLR4-deficient CD4+ T lymphocytes in presence of LPS. (**d**) Quantification of the number of activated WT CD4+ T cells stimulated with or without CLI-095. (**e**) Quantification of the number of activated WT CD4+ T cells stimulated with LPS in presence or absence of CLI-095. Two-way ANOVA with Sidak post-test was used (**a**,**c**–**e**). Data are means ± SEM from three experiments. ns = not significant, ****p < 0.0001.

To demonstrate that LPS mediates its effects through TLR4, we measured the percentage of activated TLR4-deficient CD4+ T lymphocytes exposed to increasing concentrations of anti-CD3 antibody in the absence or presence of LPS. Like their wild-type (WT) counterparts, TLR4-deficient CD4+ T lymphocytes were activated by anti-CD3 antibody in a dose-dependent manner (Fig. 4a). In contrast to WT cells, LPS did not potentiate anti-CD3-mediated activation of TLR4-deficient CD4+ T lymphocytes (Fig. 4a,b). The number of effector TLR4-deficient CD4+ T lymphocytes was also not increased by LPS at any of the anti-CD3 antibody concentrations used (Fig. 4c). Finally, the TLR4 inhibitor CLI-095, while not influencing anti-CD3-mediated activation of WT CD4+ T lymphocytes (Fig. 4d), drastically reduced LPS plus anti-CD3-mediated activation of WT CD4+ T lymphocytes (Fig. 4e). Collectively, these results strongly support the model that LPS acts through TLR4 to sensitise CD4+ T lymphocytes to anti-CD3-mediated activation.

### MIF mediates LPS-induced activation of CD4+ T lymphocytes by modulating the expression of TLR4

**Figure 5:**
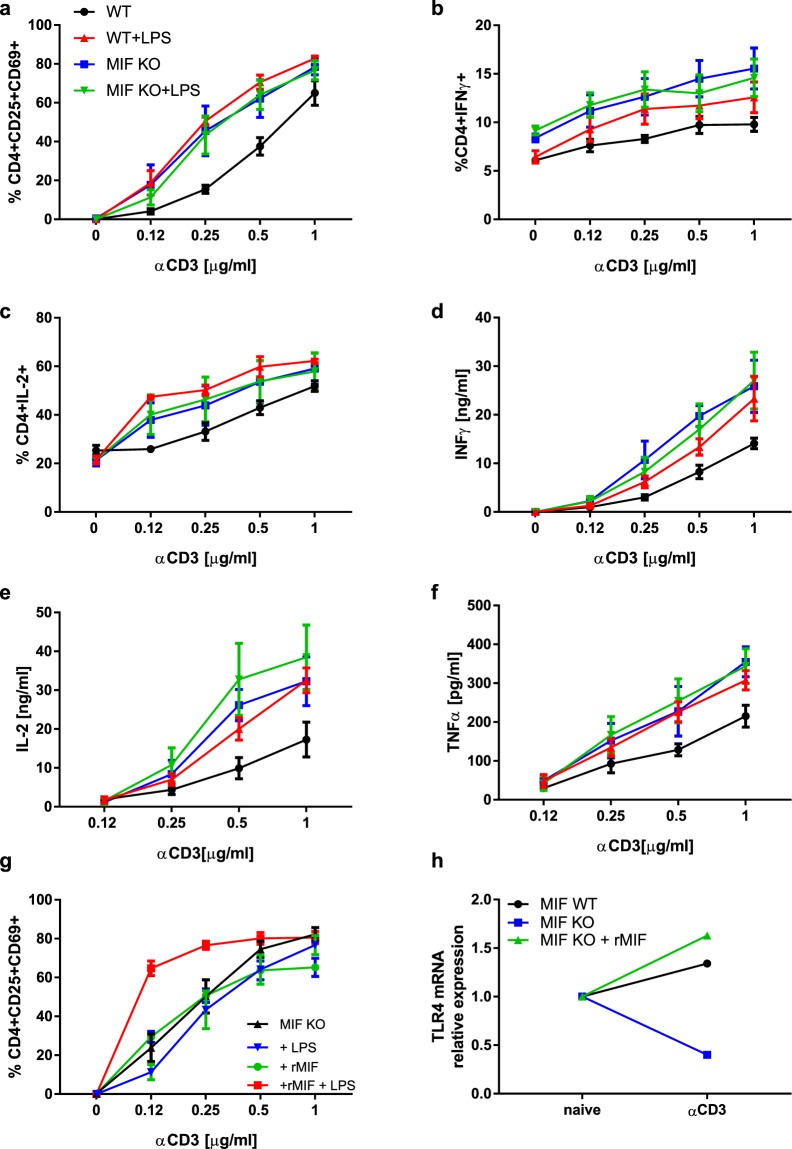
MIF mediates LPS-induced activation of CD4+ T lymphocytes by modulating the expression of TLR4 (**a**) Activation of WT and MIF-deficient CD4+ T cells in presence of LPS. Data are means ± SEM from three experiments. (**b**) Percentages of IFNγ-producing CD4+ T cells induced by LPS. Data are means ± SEM from four experiments. (**c**) Percentages of IL-2-producing CD4+ T cells. Data are means ± SEM from three experiments. (**d**) IFNγ secretion by WT and MIF-deficient CD4+ T cells in presence of LPS. Data are means ± SEM from four experiments. (**e)** IL-2 secretion by WT and MIF-deficient CD4+ T cells in presence of LPS. Data are means ± SEM from three experiments. f) TNFα secretion by WT and MIF-deficient CD4+ T cells in presence of LPS. Data are means ± SEM from five experiments. g) Activation of MIF-deficient CD4+ T cells in presence of 10 ng/ml rMIF, and LPS. Data are means ± SEM from three experiments. h) *Tlr4* mRNA expression after activation of WT and MIF-deficient CD4+ T lymphocytes: values were normalized relative to naïve cells. WT and MIF-deficient CD4+ T lymphocytes were activated with 0.5μg/ml anti-CD3 antibody. MIF-deficient CD4+ T lymphocytes were stimulated with 10 ng/ml rMIF and 0.12μg/ml anti-CD3 antibody. P > 0.05 between naïve and anti-CD3 antibody activated WT CD4+ T lymphocytes. No difference between naïve and rMIF-stimulated and anti-CD3 antibody activated MIF-deficient CD4+ T lymphocytes. p < 0.0001 between naïve and anti-CD3 antibody activated MIF-deficient CD4+ T lymphocytes. Representative of 3 different experiences. Statistical analysis of Fig. 5 is described in Table 1.

Given that MIF sustains TLR4-mediated activity of macrophages^19,22,23^, we addressed the relationship between MIF and the LPS response in CD4+ T lymphocytes. Genetic and pharmacological approaches were used to investigate whether MIF affects the response of CD4+ T lymphocytes to LPS. We first compared the responses of WT and MIF-deficient CD4+ T lymphocytes. Anti-CD3 antibody treatment increased the percentage of activated WT CD4+ T lymphocytes in a dose-dependent manner and to a greater extent that of MIF-deficient CD4+ T lymphocytes (Fig. 5a). While LPS co-stimulation further increased the percentage of activated WT CD4+ T lymphocytes, it strikingly failed to do so for MIF-deficient CD4+ T lymphocytes (Fig. 5a). In WT CD4+ T lymphocytes, LPS increased the number of effector cells (Fig. 5b,c) and their effector functions (Fig. 5d–f). In contrast, LPS increased neither the number of effector cells (Fig. 5b,c) nor their effector functions (Fig. 5d–f) in the case of MIF-deficient CD4+ T lymphocytes. There were no differences between the activation or effector functions of MIF-deficient CD4+ T lymphocytes and LPS-activated WT CD4+ T lymphocytes (Fig. 5a–f and Table 1).

**Table 1:** Statistical data of Fig. 5.

Tukey’s multiple comparisons test	Mean Diff.	95.00% CI of diff.	Significant?	Summary	Adjusted P Value
**(a) % CD4 + CD25 + CD69+**					
MIF WT vs. MIF KO	−14.66	−24.84 to −4.468	Yes	**	0.0022
MIF WT vs. MIF WT+LPS	−20.71	−30.36 to −11.05	Yes	****	<0.0001
MIF WT vs. MIF KO + LPS	−12.92	−23.11 to −2.732	Yes	**	0.0079
MIF KO vs. MIF WT + LPS	−6.05	−15.52 to 3.42	No	ns	0.3325
MIF KO vs. MIF KO + LPS	1.736	−8.275 to 11.75	No	ns	0.9666
MIF WT + LPS vs. MIF KO + LPS	7.786	−1.684 to 17.26	No	ns	0.1403
**(b) IL-2 [ng/ml]**					
MIF KO vs. MIF WT	8.042	0.9675 to 15.12	Yes	*	0.0197
MIF KO vs. MIF KO + LPS	−3.887	−12.16 to 4.388	No	ns	0.6037
MIF KO vs. MIF WT + LPS	1.165	−5.868 to 8.198	No	ns	0.9717
MIF WT vs. MIF KO + LPS	−11.93	−19 to −4.855	Yes	***	0.0002
MIF WT vs. MIF WT + LPS	−6.877	−12.45 to −1.307	Yes	**	0.0096
MIF KO + LPS vs. MIF WT + LPS	5.052	−1.981 to 12.08	No	ns	0.2399
**(c) TNFa [pg/ml]**					
MIF KO vs. MIF WT	88.67	30.07 to 147.3	Yes	***	0.0009
MIF KO vs. MIF KO + LPS	4.822	−58.07 to 67.71	No	ns	0.9971
MIF KO vs. MIF WT + LPS	16.86	−39.81 to 73.53	No	ns	0.8624
MIF WT vs. MIF KO + LPS	−83.85	−144.2 to −23.5	Yes	**	0.0027
MIF WT vs. MIF WT + LPS	−71.81	−125.7 to −17.97	Yes	**	0.0042
MIF KO + LPS vs. MIF WT + LPS	12.04	−46.44 to 70.52	No	ns	0.9486
**(d) IFNγ [ng/ml]**					
MIF KO vs. MIF WT	7.405	2.551 to 12.26	Yes	***	0.0008
MIF KO vs. MIF KO + LPS	0.9582	−4.619 to 6.535	No	ns	0.9693
MIF KO vs. MIF WT + LPS	3.081	−1.773 to 7.935	No	ns	0.3487
MIF WT vs. MIF KO + LPS	−6.447	−11.3 to −1.592	Yes	**	0.0044
MIF WT vs. MIF WT + LPS	−4.324	−8.327 to −0.3212	Yes	*	0.0291
MIF KO + LPS vs. MIF WT + LPS	2.123	−2.732 to 6.977	No	ns	0.6615
**(e) %CD4 + IFNg+**					
MIF WT vs. MIF KO	−4.129	−6.416 to −1.842	Yes	****	<0.0001
MIF WT vs. MIF WT + LPS	−1.955	−3.775 to −0.1364	Yes	*	0.03
MIF WT vs. MIF KO + LPS	−4.062	−6.349 to −1.775	Yes	****	<0.0001
MIF KO vs. MIF WT + LPS	2.174	−0.0611 to 4.408	No	ns	0.0597
MIF KO vs. MIF KO + LPS	0.067	−2.562 to 2.696	No	ns	0.9999
MIF WT + LPS vs. MIF KO + LPS	−2.107	−4.341 to 0.1281	No	ns	0.0722
**(f) % CD4 + IL-2+**					
MIF WT vs. MIF KO	−7.32	−16.45 to 1.809	No	ns	0.1553
MIF WT vs. MIF WT + LPS	−12.41	−21.54 to −3.278	Yes	**	0.0041
MIF WT vs. MIF KO + LPS	−8.107	−17.24 to 1.022	No	ns	0.0974
MIF KO vs. MIF WT + LPS	−5.087	−14.22 to 4.042	No	ns	0.4508
MIF KO vs. MIF KO + LPS	−0.7867	−9.915 to 8.342	No	ns	0.9956
MIF WT + LPS vs. MIF KO + LPS	4.3	−4.829 to 13.43	No	ns	0.5916
**(g) % CD4 + CD25+CD69 + (rMIF)**					
+rMIF vs. +rMIF + LPS	−18.59	−26.12 to −11.05	Yes	****	<0.0001
+rMIF vs. MIF KO	−3.23	−10.41 to 3.947	No	ns	0.6371
+rMIF vs. MIF KO + LPS	2.712	−5.49 to 10.91	No	ns	0.8191
+rMIF + LPS vs. MIF KO	15.36	7.754 to 22.96	Yes	****	<0.0001
+rMIF + LPS vs. MIF KO + LPS	21.3	12.72 to 29.88	Yes	****	<0.0001
MIF KO vs. MIF KO + LPS	5.942	−2.324 to 14.21	No	ns	0.2399

(a) Activation of WT and MIF-deficient CD4+ T cells in presence of LPS. Data are means ± SEM from three experiments. (b) IL-2 secretion by WT and MIF-deficient CD4+ T cells in presence of LPS. Data are means ± SEM from three experiments. (c) TNFα secretion by WT and MIF-deficient CD4+ T cells in presence of LPS. Data are means ± SEM from five experiments. (d) IFNγ secretion by WT and MIF-deficient CD4+ T cells in presence of LPS. Data are means ± SEM from four experiments. (e) Percentages of IFNγ-producing CD4+ T cells induced by LPS. Data are means ± SEM from four experiments. (f) Percentages of IL-2-producing CD4+ T cells. Data are means ± SEM from three experiments. (g) Activation of MIF-deficient CD4+ T cells in presence of 10 ng/ml rMIF, and LPS. Data are means ± SEM from three experiments. Two-way ANOVA with Tukey’s multiple comparisons test was used (a–g).

We further tested the effect of recombinant mouse MIF (rMIF) on TCR/CD3-mediated activation of MIF-deficient CD4+ T lymphocytes in the absence or presence of LPS (Fig. 5g). rMIF alone did not increase the activation of MIF-deficient CD4+ T lymphocytes (Fig. 5g). In contrast, rMIF did increase the activation of MIF-deficient CD4+ T lymphocytes in the presence of LPS.

Finally, we analysed the expression of *Tlr4* mRNA in naïve and activated MIF-deficient and WT CD4+ T lymphocytes (Fig. 5h). The expression of *Tlr4* mRNA was similar in naïve MIF-deficient and WT CD4+ T lymphocytes. The expression of *Tlr4* mRNA in WT CD4+ T lymphocytes was not affected by anti-CD3 activation. In contrast, activation decreased *Tlr4* mRNA expression in MIF-deficient CD4+ T lymphocytes, while co-incubation with rMIF restored *Tlr4* mRNA expression levels. Thus, MIF regulates the expression of TLR4 in activated CD4+ T lymphocytes, suggesting that MIF is required for TLR4-mediated activation.

### MIF regulates TCR/CD3-mediated activation of CD4+ T lymphocytes

**Figure 6:**
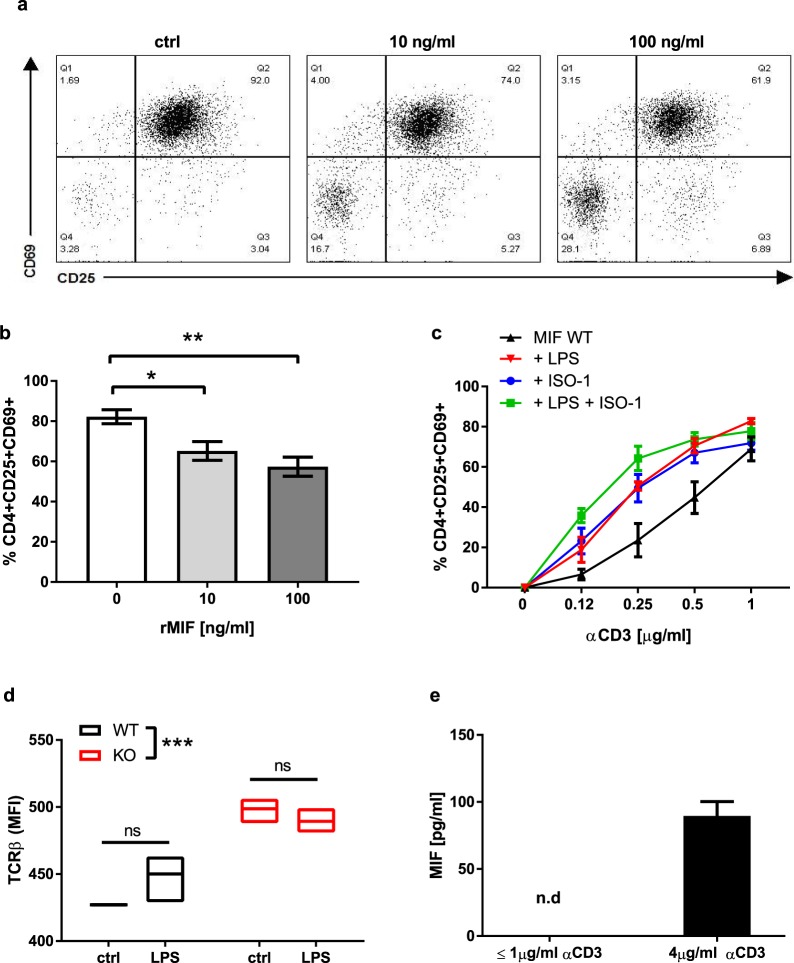
MIF regulates TCR/CD3 mediated activation. (**a**) Representative image of dose-dependent MIF-mediated inhibition of CD4+ T lymphocytes. (**b**) 1μg/ml anti-CD3-activated MIF-deficient CD4+ T cells in presence of 0, 10 and 100 ng/ml rMIF. (**c**) Activation of WT CD4+ T cells in presence of LPS and ISO-1 (p < 0.01 when WT is compared to WT+LPS, WT+ISO-1 and WT+LPS+ISO-1). (**d**) TCRβ expression in naïve WT and MIF-deficient CD4+ T cells in the presence of LPS. (**e**) MIF secretion by sub-optimally activated CD4+ T cells or after stimulation with 4 µg/ml anti-CD3 antibody. One-way ANOVA with Turkey’s post-test was used to compare variables in (**b**), and two-way ANOVA with Sidak post-test was used to compare variables in (**c**,**d**). Data are means ± SEM from three (**d**,**e**) and four experiments (b and c). *p ≤ 0.05, **p ≤ 0.01, ***p ≤ 0.001.

Compared to WT CD4+ T lymphocytes, suboptimal anti-CD3 antibody stimulation of MIF-deficient CD4+ T lymphocytes increased their activation, number and effector function to a greater extent (Fig. 5, Table 1). Accordingly, rMIF decreased TCR/CD3-mediated activation of MIF-deficient CD4+ T lymphocytes in a dose-dependent manner (Fig. 6a,b) whereas MIF inhibition with ISO-1 increased the activation of WT CD4+ T lymphocytes (Fig. 6c). Furthermore, when MIF was inhibited with ISO-1^24^, LPS did not increase the activation of WT CD4+ T lymphocytes (Fig. 6c). These results show that MIF is not only necessary for LPS-induced activation of CD4+ T lymphocytes but that it also regulates TCR/CD3-mediated activation of CD4+ T lymphocytes.

Finally, we assessed the effect of MIF on expression of the TCRβ chain, as a surrogate for TCR expression, at the surface of naïve WT and MIF-deficient CD4+ T lymphocytes, in the presence or absence of LPS (Fig. 6d). We observed that the expression of TCRβ was higher in MIF-deficient CD4+ T lymphocytes than in WT CD4+ T lymphocytes (Fig. 6d). On the other hand, TLR4 engagement by LPS did not increase the expression of TCRβ (Fig. 6d). We then evaluated the secretion of MIF by CD4+ T lymphocytes activated by anti-CD3 antibody stimulation (Fig. 6e). MIF was undetectable when the concentrations of anti-CD3 antibody were ≤1μg/ml, but was detectable only after strong anti-CD3 antibody activation (4 μg/ml) (Fig. 6e).

## Discussion

In this study, we initially demonstrated an important role of TLR4 in the adaptive immune response. We have shown that TLR4 activation in CD4+ T lymphocytes increases their activation, proliferation, differentiation and effector function. TLR4 provides a strong stimulus that enhances suboptimal anti-CD3-mediated CD4+ T lymphocyte activation. These data demonstrate that TLR4 expressed by CD4+ T lymphocytes is functional and able to modulate their responses.

Interestingly, we observed no effect of LPS alone on CD4+ T lymphocyte activation or effector function. To have an effect on the activation and effector functions of CD4+ T lymphocytes, the induction of TLR4 signalling requires suboptimal anti-CD3-mediated activation. These observations suggest that there is a crosstalk between TLR4 and TCR/CD3 signalling. This crosstalk has also been observed for other TLRs expressed by CD4+ T lymphocytes. Functional effect elicited by the activation of TLR2, TLR3, TLR5 and TLR9 were also found to be dependent on TCR/CD3 signalling^5,7,25–27^.

Until now, studies on the effects of LPS on CD4+ T lymphocyte responses have focused mainly on the impact of LPS on APCs^28^. Two previous studies on the role of TLR4 activation in CD4+ T lymphocytes led to contradictory interpretations, probably because different methods and readouts were used, despite the fact that both used TLR4-deficient lymphocytes^1,2^. In addition, other signals could interfere with lymphocyte response *in vivo*. One report highlighted a protective role of TLR4 activation in CD4+ T lymphocytes, whereas a second demonstrated a pathogenic role. These conflicting findings highlight the importance of the microenvironment and its impact on the activation of T lymphocytes and the pathogenesis of inflammatory and autoimmune diseases. Discrepancies may also result from the experimental models used, namely focusing on the central nervous system or the gut. In an environment rich in microorganisms such as the gut, TLR4 signalling could be beneficial for alarming the host, while in a sterile environment such as the CNS, TLR4 activation could lead to deleterious neuro-inflammation. In this context, TLR4 as a sensor of the microenvironment contributes to the initiation and maintenance of autoimmune or inflammatory processes.

We demonstrated that MIF, a preformed cytokine secreted by T lymphocytes after anti-CD3-mediated activation, and an important regulator of the expression of TLR4 in macrophages^11,22^, is indispensable for LPS-induced activation of CD4+ T lymphocytes. It has been observed that MIF-deficient macrophages exhibit decreased expression of TLR4 and secrete less pro-inflammatory cytokines. Although we did not observe decreased TLR4 expression in naïve MIF-deficient CD4+ T lymphocytes, it was reduced in activated MIF-deficient CD4+ T lymphocytes. Adding rMIF was able to restore the expression of TLR4 in activated MIF-deficient CD4+ T lymphocytes. Therefore, MIF seems to maintain the expression of TLR4 in activated CD4+ T lymphocytes, thereby enabling their response to LPS stimulation. The absence of a difference in basal TLR4 expression in WT and MIF-deficient CD4+ T lymphocytes implies that TLR4 is expressed constitutively in naïve T lymphocytes. This result corroborates previous observations on the pattern of TLR4 expression by T lymphocytes, which indicated that TLR4 is expressed on both naïve and activated CD4+ T lymphocytes^1–3,5–7^.

LPS increased neither the number of MIF-deficient CD4+ T lymphocytes nor their effector functions, probably because of reduced TLR4 expression by activated MIF-deficient CD4+ T lymphocytes compared to WT CD4+ T lymphocytes. In this regard, the addition of rMIF both maintained the level of TLR4 expression in MIF-deficient CD4+ T lymphocytes and enabled stronger LPS-induced activation of these cells.

**Figure 7:**
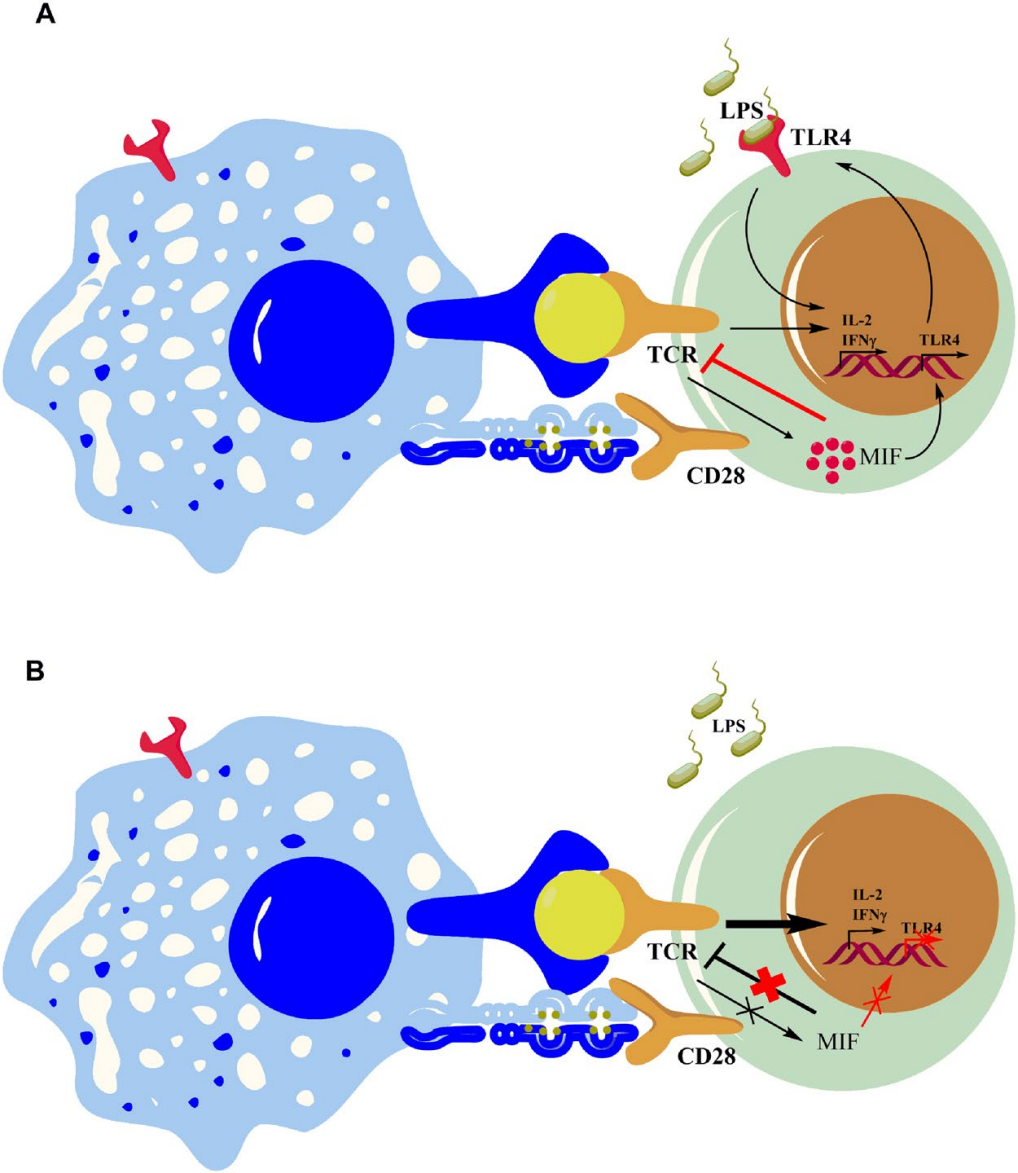
Schematic representation of the role of MIF in CD4+ T lymphocyte response. (**A**) Engagement of the TCR/CD3 complex induces T cell activation and adaptive immune responses. MIF negatively regulates suboptimal TCR/CD3-mediated activation and induces the expression of TLR4. Engagement of TLR4 by LPS enhances TCR-mediated activation and enables stronger activation in presence of LPS, despite suboptimal TCR activation. In the presence of a strong TCR/CD3 signal, MIF is secreted, thus abrogating TCR-inhibition. (**B**) In the absence of MIF, sub-optimal TCR-stimulation induces supra-optimal activation and an excessive effector response. TLR4 expression decreases after TCR/CD3-mediated activation in MIF-deficient CD4+ T lymphocytes.

We observed that MIF regulates the expression of both TLR4 and the TCR/CD3 complex. Indeed, WT CD4+ T lymphocytes express less TCRβ than MIF-deficient CD4+ T lymphocytes. In addition, WT CD4+ T lymphocytes are less activated than MIF-deficient CD4+ T lymphocytes upon suboptimal TCR/CD3 stimulation. We confirmed the regulatory role of MIF in TCR/CD3-mediated activation using the MIF inhibitor ISO-1. Reminiscently of what was observed in MIF-deficient CD4+ T lymphocytes, ISO-1 both increased TCR/CD3-mediated activation of WT CD4+ T lymphocytes and abrogated the effect of LPS on these cells. Moreover, exposure to rMIF induced a dose-dependent reduction in TCR/CD3-mediated activation of MIF-deficient CD4+ T lymphocytes. In addition, MIF was not detected when cells were stimulated with less than ≤1μg/ml anti-CD3 antibodies, concentrations at which the inhibitory effect of MIF was maximal. This is consistent with the previous observation by Bacher et *al*., that MIF is secreted only in presence of strong anti-CD3 activation^29^. Taken together, our results suggest that MIF could be a major regulator and rheostat of TCR/CD3-mediated activation of CD4+ T lymphocytes when antigen concentrations are suboptimal (Fig. 7).

MIF functions as a counter-regulator of the immunosuppressive effect glucocorticoids^29,30^. Bacher *et al*. observed that rMIF counter-acted dexamethasone-induced inhibition of pro-inflammatory cytokine production^29^ and that, in contrast to our observation, MIF neutralizing antibodies inhibited the proliferation of T lymphocytes and the secretion of pro-inflammatory cytokines^29^. Of note, this effect was obtained when T lymphocytes were stimulated with a concentration of anti-CD3 antibody that was 5 times higher than the maximum concentration used here. Surprisingly, no pro-inflammatory response was noticeable when they used rMIF *in vitro* or *in vivo*. Calandra *et al*. also reported that MIF neutralizing antibodies inhibited the proliferation of mouse splenocytes induced by the toxic-shock syndrome toxin 1 (TSST1) and protected mice from TSST1-induced shock^31^. However, apparently contradictory observations have just been reported^32^. Kim *et al*. showed that γδ, but not αβ T lymphocytes from MIF-deficient mice produced increased levels of IL-17A, and that MIF-deficient mice were more susceptible to toxic shock. Altogether, our observations combined with and those published previously suggest that MIF, rather than being a direct mitogenic agent, might be a direct mediator of T cell responses. Its function in T cell activation appears to be dependent on the strength of the activation signal, and possibly on the subpopulation of T lymphocytes.

In accordance with our observations, Yan *et al*. observed that a cancer cell line overproducing MIF was able to inhibit the activation of T lymphocytes induced by anti-CD3 antibody treatment as well as by IL-2 and IL-15^33^. It was concluded that IFNγ mediated the inhibition of T lymphocytes by MIF. In agreement with these findings, we have confirmed that MIF inhibits the activation of T lymphocytes. We have further shown that this effect of MIF occurs through its regulation of the TCR/CD3 complex. In this context, the difference in the secretion of IFNγ is a consequence of the regulation of the TCR/CD3 complex. Nevertheless, the effect of MIF can vary depending on its concentration and target cells^30^. Differences observed between the results of Bacher et *al*., Yan et *al*. and our study could be related to differences in the population of T lymphocytes studied (pan-T cells in Bacher et *al*. and Yan et *al*. *vs* purified CD4+ T cells in our experiments), and to the mode or efficacy of T lymphocyte activation (anti-CD3/28 by Bacher et *al*. and our experiments versus IL-2 and IL-15 by Yan *et al*.).

In conclusion, by exploring MIF function using both pharmacological agents (rMIF and ISO-1) and MIF-deficient mice we were able to describe crucial roles of MIF in the activation of CD4+ T lymphocytes. Interactions between MIF, TLR4 and TCR/CD3 are complex and understanding their nature could have translational impacts on immunomodulatory approaches for treating inflammatory and autoimmune diseases.

## Methods

### Materials

Materials used in the manuscript are listed in Supplementary Table S1

### Animals

All mice, described in the material section, were maintained under specific pathogen-free conditions and all animal experiments were performed in accordance with the Guidelines for the Care and Use of Laboratory Animals and approved by the Animal experimentation & Animal Welfare Office of the University of Geneva and the Geneva Veterinary Authorities.

### Cell culture reagents

Spleen lymphocytes were cultured in RPMI 1640 (Life Technologies) supplemented with 10% heat-inactivated FCS (Biochrom GmbH), 1 mM HEPES Buffer Solution (Life Technologies), 25 mM sodium pyruvate solution (Sigma), 0.5 mM 2-mercaptoethanol (Bio-Rad), MEM Non-essential Amino Acid Solution 1 × (Sigma), 100 U/ml Penicillin and 100 U/μg/ml Streptomycin (Life Technologies).

### *In vitro* T lymphocyte activation

Spleens from mice were harvested and single-cell suspensions were prepared by crushing spleens on 70 μm cell strainers. Mouse T lymphocytes and CD4+ T lymphocytes were purified from splenocytes with the Pan T Cell Isolation Kit II (Miltenyi Biotec) and a CD4^+^ T Cell Isolation Kit (Miltenyi Biotec) following manufacturer’s instructions. Purity was between 98.5–99%. Purified cells (2 × 10^5^) were cultured for 48 h at 37° with various concentrations of anti-CD3 antibody, 1 μg/ml anti-CD28, in presence or absence of 1 μg/ml ultrapure LPS, 10 μM CLI-095, 50 μM ISO-1 or various concentrations of rMIF. For the proliferation assay, lymphocytes were stained with 2.5 μM CFSE (Life Technologies) following the manufacturer’s instructions and stimulated with different concentrations of LEAF anti-CD3 antibody, 1 μg/ml anti-CD28 antibody and 1 μg/ml ultrapure *E.coli* O111:B4 LPS for 48 h. T lymphocyte activation, proliferation and differentiation were assessed by FACS, using Cyan ADP (Beckman Coulter). Data were analyzed with FlowJo software. Proliferation was analyzed with FlowJo’s proliferation tool: the division index refers to the average number of cell division in the total cell population and the proliferation index refers to the average number of cell division in the dividing cell population

### Flow cytometry

Lymphocytes were stained with fluorochrome-conjugated antibodies following the manufacturer’s recommended usage. Intracellular staining was performed after stimulation with the Cell Activation Cocktail (Biolegend) for 4 h at 37 °C. Cells were then fixed and permeabilized using the Intracellular Fixation & Permeabilization Buffer Set (eBioscience) and staining was performed. For both surface staining and intracellular staining the Zombie Red™ Fixable Viability Kit (Biolegend) was used to exclude dead cells.

### ELISA and ELISPOT

IFNγ, TNFα and IL-2 quantifications were performed on cell culture supernatants with Ready-SET-Go! kits (eBioscience) according to manufacturer’s instructions. MIF was quantified by ELISA (Biomatik) using 48 h cell culture supernatants from naïve and anti-CD3 antibody-activated CD4+ T lymphocytes. Two hundred thousand lymphocytes were seeded in MultiScreen-IP 96-wells (Merck Millipore), pre-coated with mouse IFNγ from ELISPOT Ready-SET-Go (eBiosciences) in the presence or absence of LPS for 24 h. ELISPOT membranes were revealed following the manufacturer’s instructions and data was acquired and analyzed with the ImmunoSpot analyzer (CTL Europe GmbH).

### Real-time quantitative PCR analysis

RNA was extracted from 5–10^6^ naïve and anti-CD3 antibody-activated CD4+ T lymphocytes using PeqGold Trifast (PeqLab). RNA was reverse transcribed using the High Capacity cDNA Reverse transcription kit (ThermoFischer Scientific). Gene amplification was achieved by using the TaqMan Fast Advance Master Mix (ThermoFischer Scientific). Primers used for amplification were purchased from ThermoFischer Scientific: mouse Rplp0 (Mm99999223_gH) and mouse Tlr4 (Mm00445273_m1). Tlr4 gene expression in activated CD4+ T lymphocytes was normalized to the value observed for naïve cells and calculated using the comparative cycle threshold Ct method (2^−ΔCt^ method).

### Statistical analysis

GraphPad Prism was used for all statistical analyses. Student *t*-test was used to compare two groups, one-way ANOVA with Turkey’s post-test was used to compare one variable in more than two groups, and two-way ANOVA with Sidak post-test was used to compare 2 variables in more than 2 groups. Differences between the groups were considered to be significative when p ≤ 0.05. Data is presented with the mean ± standard error of mean (s.e.m).

## Supplementary information

Supplementary figures

## Data Availability

The datasets produced in this study will be made available upon reasonable request. Requests should be sent to the corresponding author.

## References

[1] González-Navajas JM (2010). TLR4 signaling in effector CD4+ T cells regulates TCR activation and experimental colitis in mice. J. Clin. Invest..

[2] Reynolds JM, Martinez GJ, Chung Y, Dong C (2012). Toll-like receptor 4 signaling in T cells promotes autoimmune inflammation. Proc. Natl. Acad. Sci. USA.

[3] Caramalho I (2003). Regulatory T cells selectively express toll-like receptors and are activated by lipopolysaccharide. J. Exp. Med..

[4] Tomita T (2008). MyD88-Dependent Pathway in T Cells Directly Modulates the Expansion of Colitogenic CD4+ T Cells in Chronic Colitis. J. Immunol..

[5] Gelman AE, Zhang J, Choi Y, Turka LA (2004). Toll-like receptor ligands directly promote activated CD4+ T cell survival. *J. Immunol. Baltim*. Md 1950.

[6] Fukata M (2008). The Myeloid Differentiation Factor 88 (MyD88) Is Required for CD4+ T Cell Effector Function in a Murine Model of Inflammatory Bowel Disease. J. Immunol..

[7] Komai-Koma M, Jones L, Ogg GS, Xu D, Liew FY (2004). TLR2 is expressed on activated T cells as a costimulatory receptor. Proc. Natl. Acad. Sci. USA.

[8] Zanin-Zhorov A (2007). Cutting Edge: T Cells Respond to Lipopolysaccharide Innately via TLR4 Signaling. J. Immunol..

[9] Rahman AH, Taylor DK, Turka LA (2009). The contribution of direct TLR signaling to T cell responses. Immunol. Res..

[10] Bloom BR, Bennett B (1966). Mechanism of a reaction *in vitro* associated with delayed-type hypersensitivity. Science.

[11] Calandra T, Roger T (2003). Macrophage migration inhibitory factor: a regulator of innate immunity. Nat. Rev. Immunol..

[12] Bernhagen J (2007). MIF is a noncognate ligand of CXC chemokine receptors in inflammatory and atherogenic cell recruitment. Nat. Med..

[13] Leng L (2003). MIF signal transduction initiated by binding to CD74. J. Exp. Med..

[14] Shi X (2006). CD44 is the signaling component of the macrophage migration inhibitory factor-CD74 receptor complex. Immunity.

[15] Filip A-M (2009). Ribosomal protein S19 interacts with macrophage migration inhibitory factor and attenuates its pro-inflammatory function. J. Biol. Chem..

[16] Hudson JD (1999). A proinflammatory cytokine inhibits p53 tumor suppressor activity. J. Exp. Med..

[17] Kleemann R (2000). Intracellular action of the cytokine MIF to modulate AP-1 activity and the cell cycle through Jab1. Nature.

[18] Roger T, Chanson A-L, Knaup-Reymond M, Calandra T (2005). Macrophage migration inhibitory factor promotes innate immune responses by suppressing glucocorticoid-induced expression of mitogen-activated protein kinase phosphatase-1. Eur. J. Immunol..

[19] Roger T, Froidevaux C, Martin C, Calandra T (2003). Macrophage migration inhibitory factor (MIF) regulates host responses to endotoxin through modulation of Toll-like receptor 4 (TLR4). J. Endotoxin Res..

[20] Calandra T, Roger T (2003). Macrophage migration inhibitory factor: a regulator of innate immunity. Nat. Rev. Immunol..

[21] Bucala R (2013). MIF, MIF alleles, and prospects for therapeutic intervention in autoimmunity. J. Clin. Immunol..

[22] Roger T, David J, Glauser MP, Calandra T (2001). MIF regulates innate immune responses through modulation of Toll-like receptor 4. Nature.

[23] Roger T (2013). Macrophage Migration Inhibitory Factor Deficiency Is Associated With Impaired Killing of Gram-Negative Bacteria by Macrophages and Increased Susceptibility to Klebsiella pneumoniae Sepsis. J. Infect. Dis..

[24] Al-Abed Y (2005). ISO-1 Binding to the Tautomerase Active Site of MIF Inhibits Its Pro-inflammatory Activity and Increases Survival in Severe Sepsis. J. Biol. Chem..

[25] Reynolds JM (2010). Toll-like Receptor 2 Signaling in CD4+ T Lymphocytes Promotes T Helper 17 Responses and Regulates the Pathogenesis of Autoimmune Disease. Immunity.

[26] Liu H, Komai-Koma M, Xu D, Liew FY (2006). Toll-like receptor 2 signaling modulates the functions of CD4+CD25+ regulatory T cells. Proc. Natl. Acad. Sci..

[27] Crellin NK (2005). Human CD4^+^ T Cells Express TLR5 and Its Ligand Flagellin Enhances the Suppressive Capacity and Expression of FOXP3 in CD4^+^CD25^+^ T Regulatory Cells. J. Immunol..

[28] Mills KHG (2011). TLR-dependent T cell activation in autoimmunity. Nat. Rev. Immunol..

[29] Bacher M (1996). An essential regulatory role for macrophage migration inhibitory factor in T-cell activation. Proc. Natl. Acad. Sci..

[30] Calandra T (1995). MIF as a glucocorticoid-induced modulator of cytokine production. Nature.

[31] Calandra T, Spiegel LA, Metz CN, Bucala R (1998). Macrophage migration inhibitory factor is a critical mediator of the activation of immune cells by exotoxins of Gram-positive bacteria. Proc. Natl. Acad. Sci. USA.

[32] Kim Hee Kyung, Garcia Alvaro Baeza, Siu Edwin, Tilstam Pathricia, Das Rita, Roberts Scott, Leng Lin, Bucala Richard (2019). Macrophage migration inhibitory factor regulates innate γδ T-cell responses via IL-17 expression. The FASEB Journal.

[33] Yan X, Orentas RJ, Johnson BD (2006). Tumor-derived macrophage migration inhibitory factor (MIF) inhibits T lymphocyte activation. Cytokine.

