# Supplementary material for: Macrophage migration inhibitory factor regulates TLR4 expression and modulates TCR/CD3-mediated activation in CD4+ T lymphocytes
Source: Sci Rep. 2019 Jun 28;9:9380. doi: 10.1038/s41598-019-45260-6 (PMC6599059; doi:10.1038/s41598-019-45260-6)
Supplement: Supplementary file 1 — Supplementary figures [file 41598_2019_45260_MOESM1_ESM.pdf]

Macrophage migration inhibitory factor regulates TLR4 expression and modulates TCR/CD3-mediated activation in CD4+ T lymphocytes

Mohamed Alibashe-Ahmed, Thierry Roger, Veronique Serre-Beinier, Ekaterine Berishvili, Walter Reith, Domenico Bosco, and Thierry Berney

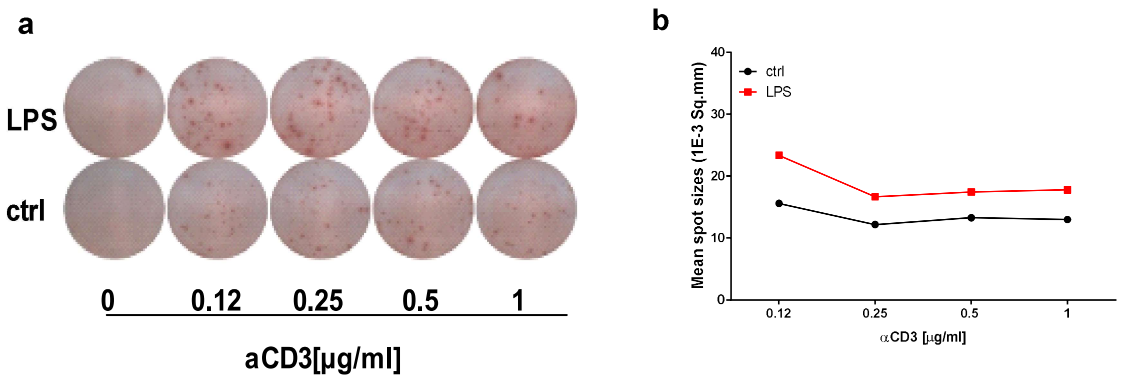

Figure S1: LPS increases the number of IFNγ-producing T lymphocytes a) Representative images of IFNγ-ELISPOT of CD3+ T lymphocytes stimulated with LPS b) Qualitative measurement of IFNγ production represented by IFNγ-ELISPOT mean spot size in presence (red) or absence (black) of LPS Data are means of five experiments (b)

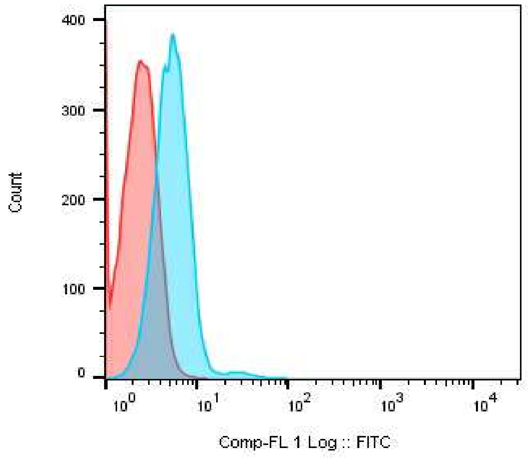

Figure S2: LPS directly interacts with CD4+ T lymphocytes. Representative image of purified CD4+ T lymphocytes stained with FITC-labelled-LPS (blue) and without (red).

REAGENT or RESOURCE	SOURCE	IDENTIFIER
**Antibodies**		
low endotoxin azide free (LEAF) purified anti-mouse CD3ε antibody (145-2C11)	Biolegend	100314
low endotoxin azide free (LEAF) purified anti-mouse CD3ε antibody (17A2)	Biolegend	100208
Pure anti-CD28 (clone 37.51)	Milteyni Biotec	130-093-182
Mouse Pan T Cell Isolation Kit II	Miltenyi Biotec	130-095-130
Mouse $CD4^+$ T Cell Isolation Kit	Miltenyi Biotec	130-104-454
FITC anti-mouse CD25 Antibody (3C7)	Biolegend	101908
PE/Cy7 anti-mouse CD69 Antibody (H1.2F3)	Biolegend	104512
PE anti-mouse CD69 Antibody (H1.2F3)	Biolegend	104508
PE/Cy7 anti-mouse CD8a Antibody (53-6.7)	Biolegend	100708
PE anti-mouse CD8a Antibody (53-6.7)	Biolegend	100722
APC/Cy7 anti-mouse CD8a Antibody (53-6.7)	Biolegend	100714
APC anti-mouse CD8a Antibody (53-6.7)	Biolegend	100711
PerCP anti-mouse CD4 Antibody (RM4-5)	Biolegend	100537
PE/Dazzle™ 594 anti-mouse CD4 Antibody (GK1.5)	Biolegend	10455
PE anti-mouse CD3 Antibody (17A2)	Biolegend	100205
FITC anti-mouse IFN-γ Antibody (XMG1.2)	Biolegend	505806
PE anti-mouse IL-2 (JES6-5H4)	Biolegend	503807
FITC Rat IgG1 κ Isotype Ctrl Antibody (RTK2071)	Biolegend	400405
PE Rat IgG2b κ Isotype Ctrl Antibody (RTK4530)	Biolegend	400607
FITC Rat IgG2b κ Isotype Ctrl Antibody (RTK4530)	Biolegend	400634
PE Rat IgG2a κ Isotype Ctrl Antibody (RTK2758)	Biolegend	400508
PE Armenian Hamster IgG Isotype Ctrl Antibody (HTK888)	Biolegend	400907
PE/Cy7 Rat IgG1 κ Isotype Ctrl Antibody (RTK2071)	Biolegend	400416
Zombie Red™ Fixable Viability Kit (	Biolegend	423110
**Bacterial and Virus Strains**		
ultrapure LPS from *E. coli O111:B4*	Invivogen	Tlrl-3pelps
ultrapure LPS from *P.gingivalis*	Invivogen	Tlrl-ppglps
ultrapure LPS from *S. minnesota* R595	Invivogen	Tlrl-smlps
FITC-conjugated Lipopolysaccharides from *Escherichia coli* O111:B4	Sigma-Aldrich	F3665-1MG
**Chemicals, Peptides, and Recombinant Proteins**		
CLI-095	Invivogen	Tlrl-cli95
CFSE	Life Technologies	C34554
recombinant mouse MIF	Biolegend	
ISO-1	Merck Millipore	475837
**Critical Commercial Assays**		
Cell Activation Cocktail	Biolegend	423303
Intracellular Fixation & Permeabilization Buffer Set	eBioscience	88-8824-00

Mouse IL-2 Ready-SET-Go! kits	Thermofischer	88-7024-22
Mouse IFNγ Ready-SET-Go! kits	Thermofischer	88-7314-22
Mouse TNFα Ready-SET-Go! kits	Thermofischer	88-7324-22
Mouse IFNγ from ELISPOT Ready-SET-Go	Thermofischer	88-7384-21
Mouse MIF ELISA kit	Biomatik	EKU05762
High Capacity cDNA Reverse transcription kit	ThermoFischer Scientific	4368814
PeqGold Trifast	PeqLab	30-2010
TaqMan Fast Advance Master Mix	ThermoFischer Scientific	4444556
**Experimental Models: Organisms/Strains**		
Mouse NOD/BDC2.5 NOD.CgTg(TcraBDC2.5,TcrbBDC2.5)1Doi/DoiJ (NOD/BDC2.5)	Jackson Laboratory	004460
Mouse TLR4$^{-/-}$ B6.B10ScN-Tlr4lps-del/JthJ	Jackson Laboratory	007227
Mouse TLR4$^{+/+}$ C57BL/6J	Jackson Laboratory	000664
Mouse MIF$^{-/-}$	John David (Harvard Medical School, Boston, MA)	
Mouse MIF$^{+/+}$	John David (Harvard Medical School, Boston, MA)	
**Oligonucleotides**		
Primer mouse Rplp0 (Mm99999223_gH)	ThermoFischer Scientific	4331182
Primer mouse Tlr4 (Mm00445273_m1)	ThermoFischer Scientific	4331182
**Software and Algorithms**		
GraphPad Prism 7	GraphPad Software	
FlowJo V_10	FlowJo, LLC	

Supplementary Table S1: list of materials.
